# Optimization of Thiolated Chitosan Nanoparticles for the Enhancement of in Vivo Hypoglycemic Efficacy of Sitagliptin in Streptozotocin-Induced Diabetic Rats

**DOI:** 10.3390/pharmaceutics12040300

**Published:** 2020-03-26

**Authors:** Kousalya Prabahar, Ubaidulla Udhumansha, Mona Qushawy

**Affiliations:** 1 Department of Pharmacy Practice, Faculty of Pharmacy, University of Tabuk, Tabuk 71491, Saudi Arabia; 2 Department of Pharmaceutics, C.L.Baid Metha College of Pharmacy, Chennai 600097, India; ubaidnkl@gmail.com; 3 Department of Pharmaceutics, Faculty of Pharmacy, University of Tabuk, Tabuk 71491, Saudi Arabia; mqushawy@ut.edu.sa; 4 Department of Pharmaceutics, Faculty of Pharmacy, Sinai University, Alarish, North Sinai 45511, Egypt

**Keywords:** Sitagliptin, thiolated chitosan (TC), ionic gelation, hypoglycemic activity, Box-Behnken design, mucoadhesion study

## Abstract

Sitagliptin (SGN) is an antidiabetic drug used for treatment of diabetes mellitus type II. The objectives of this study were to formulate SGN in form of thiolated chitosan (TC) nanoparticles to enhance the mucoadhesion properties of SGN to the gastrointestinal tract, prolong drug release, decrease side effects, and enhance patient compliance. Seventeen batches of SGN-TC nanoparticles were designed by Box-Behnken design and prepared using the ionic gelation method using tripolyphosphate (TPP) as crosslinking agent. The prepared formulations were evaluated for particle size, entrapment efficiency %, and in vitro drug release. Based on the results of optimization, three formulations (F1–F3) were prepared with different drug polymer ratios (1:1, 1:2, and 1:3). The mucoadhesion study and in vivo hypoglycemic activity of three formulations were evaluated in comparison to free SGN in streptozotocin (STZ)-induced diabetic rats. The seventeen SGN-TC nanoparticles showed small particle sizes, high entrapment efficiency, and prolonged drug release. The concentration of TC polymers had highest effect on these responses. The percentage of SGN–TC nanoparticles adhered to tissue was increased and the release was prolonged as the concentration of TC polymer increased (F3 > F2 > F1). The hypoglycemic effect of SGN-TC nanoparticles was significantly higher than resulted by free SGN. It was concluded that TC nanoparticles had the ability to enhance the mucoadhesion properties of SGN and prolong the drug release. SGN-TC nanoparticles significantly reduced plasma glucose levels compared to free SGN in STZ-induced diabetic rats.

## 1. Introduction

The mucoadhesive delivery system has improved the pharmacokinetic and pharmacological properties of drugs, which can be advantageous when used in the treatment of acute and chronic diseases [1]. The mucoadhesive drug delivery carrier has many potential advantages, like improved bioavailability of drugs, due to increasing the residence time in the mucosa, a lower frequency of administration by controlling drug release in the gastrointestinal tract, and target specificity to a particular site by placing drugs directly into the mucosal tract [2,3]. The literature signifies that different polymers, either natural or synthetic, have gained considerable interest in the pharmaceutical industry and academic research due to this mucoadhesive property that can deliver the oral dosage efficiently [4].

Recently, it has been shown that higher adhesive properties can be provided by polymers containing thiol groups which showed enhanced absorption of the drug in the gastro intestinal tract (GIT) region [5]. The new generation of mucoadhesive polymers is strong enough to form covalent bonds to the mucus layer; this is unlike the first generation polymers which are less interactive with the mucus layer through formation of a non-covalent bond [6]. Thiolated polymers, which are also covalently adhered to the mucus layer through formation of disulfide bridges with the glycoproteins of mucin, result in enhanced mucoadhesion [7]. Chen et al. and Shahnaz et al. reported that thiolated polymers could improve mucoadhesive properties up to 140-fold due to the immobilization mechanism of thiol groups [8,9].

Diabetes mellitus is the most common metabolic disorder of the human population. It is global in distribution, affecting 2% to 6% of the world population [10]. Diabetes mellitus may be a chronic illness, and therefore the period of treatment medical aid is the patient’s lifespan. In recent years, sustained release formulations of antidiabetic drugs have received much attention due to improved patient compliance by reducing the dosage regimen, reduction in the amount of dose, reduced gastrointestinal side effects (such as diarrhea, abdominal discomfort, and flatulence), and improved patient adherence to the simple dosing regimen [11].

Sitagliptin (SGN), a dipeptidyl peptidase-4 (DPP-4) enzyme inhibitor, has been used for the management of glycemic control in type II diabetes mellitus [12]. The pharmacokinetics of SGN after single-dose administration by oral therapy in a healthy human population shows rapid absorption with an absolute bioavailability of ~87% and C_max_ (peak plasma concentrations) reached in 1–4 h [13]. Almost 79% of SGN is excreted unchanged in urine, which lead to administration of larger doses to maintain the therapeutically effective plasma level of the drug [14]. Moreover, the frequent administration of the drug might bring a decrease in the patient compliance. To overcome these limitations, nanoparticle formulations are being developed to prolong drug release in a well-controlled manner and hence improve patient compliance.

Chitosan is a natural, cationic polysaccharide [15]. It has received a great deal of attention as a completely unique excipient in preparation of nanoparticles due to its favorable properties. Chitosan nanoparticles can be used for management of several diseases as cancer, pulmonary diseases, gastrointestinal disorders, and ocular infections [16]. Chitosan has several other useful characteristics such as being biodegradable, non-toxic, and biocompatible [17]. The presence of an amino group in chitosan increases its ability to undergo a simple chemical modifications [18]. Chitosan possesses inherent mucoadhesive properties which may be beneficial in developing mucoadhesive preparations. The mucoadhesive properties of chitosan can be increased by attaching a thiol (-SH-) containing side groups to its backbone chains through ionic gelation with thioglycolic acid [19]. Earlier reports revealed that the nanoparticles prepared using thiolated chitosan (TC) polymer are expected to have strong cohesive properties with the mucus layer, making them extremely appropriate excipients for developing prolonged release dosage forms [20]. Chen et al. prepared chitosan nanoparticles to enhance the transport of dextran and curcumin through intestinal epithelial cell layer [21]. Based on the hypothesis, the present work is aimed to develop sitagliptin entrapped thiolated chitosan nanoparticles (SGN–TC) using the ionic gelation technique to achieve uniform size distribution of particles, good entrapment efficiency, and a prolonged drug release profile from TC nanoparticles. The in vivo hypoglycemic efficacy of the SGN was investigated using streptozotocin (STZ) induced diabetes rat model.

## 2. Materials and Methods 

### 2.1. Materials

Sitagliptin, chitosan (low molecular weight, 100,000 Da and 75–85% deacetylated) and thioglycolic acid were purchased from Sigma–Aldrich Co. (St. Louis, MO, USA). Other chemicals were of analytical grade. Double-distilled deionized water was used for the experiments.

### 2.2. Methods

#### 2.2.1. Experimental Design

A Box-Behnken design was used to determine the effect of three factors, concentration of TC (A; X_1_), concentration of the cross-linking agent, tripolyphosphate, TPP (B; X_2_), and concentration of SGN (C; X_3_), on the responses, Y_1_ (particle size nm), Y_2_ (entrapment efficiency %), and Y_3_ (drug release %). 

#### 2.2.2. Preparation of SGN–TC nanoparticles

Thiolated chitosan (TC) was prepared by immobilization of amide bonds between the amino groups of chitosan polymer and the carboxylic acid groups of thioglycolic acid (TGA) as described earlier by Martien et al. [22]. SGN–TC nanoparticles were prepared by ionic gelation between positively charged amino group of TC and negatively charged phosphate ions of cross-linking agent (TPP) [23]. Briefly, TC was dissolved in aqueous solution of acetic acid (2% *w*/*w*) then allowed to stir overnight at 25 °C. The pH of the resultant solution was converted from 4 to 5 by addition of a minimum amount of NaOH (2 M) solution. Afterwards, an accurate amount of SGN was homogenized with polymeric solution using a homogenizer (Remi Motors, Mumbai, India). The aqueous solution of TPP was prepared using distilled water and stored by cooling in a fridge at 0–2 °C for 4 h. The solution of TC was stirred for 10 min in a preheated water bath at 60 °C. After that, the solution of TC containing SGN was transferred to an ice bath and the aqueous solution of TPP was added to the mixture with continuous stirring for 10 min [24]. The stirring was continued for a further 15 min after removal from ice bath until opalescent suspension of SGN–TC nanoparticles was obtained. The prepared SGN-TC nanoparticles were subjected to centrifugation at 3000 rpm for 30 min then stored in well closed container for further use during the study.

#### 2.2.3. Particle Size Analysis (Y_1_)

The particle size of SGN-TC nanoparticles was measured using the laser light scattering technique using a Malvern zeta sizer (Malvern Master Sizer 2000, SM, Malvern, United Kingdom). Samples of SGN–TC nanoparticles were stirred in the sample unit after dilution with double distilled water [25,26,27]. The laser obscuration range was maintained in the range of 15%–20%. The experiment was done in triplicate and mean ± SD was measured. 

#### 2.2.4. Determination of Entrapment efficiency % (Y_2_)

The entrapment efficiency of all prepared SGN–TC nanoparticles was measured by a direct method. Accurate amounts of each nanoparticle formulation were soaked in 50 mL of double distilled water for about 30 min, and then subjected to sonication using a probe sonicator for 15 min to allow the breakage of nanoparticles and release of the entrapped drug. After sonication, each sample was subjected to centrifugation to separate the remaining polymeric debris from the supernatant. The remaining amount of the drug was separated by washing the polymeric debris with fresh water [28]. The collected supernatant was subjected to spectrophotometric analysis to determine the amount of SGN at *λ*_max_ of 265 nm. The entrapment efficiency for all prepared SGN–TC nanoparticles was measured in triplicate and mean ± SD was measured using the following Equation (1):

(1) $$\mathrm{Entrapment}\;\mathrm{effeciancy}\;\left(\mathrm{EE}\%\right)=\frac{\mathrm{percentage}\;\mathrm{drug}\;\mathrm{loading}}{\mathrm{thioretical}\;\mathrm{drug}\;\mathrm{loading}}\times 100$$

#### 2.2.5. In Vitro Drug Release Studies (Y_3_)

The in vitro release study for the prepared SGN–TC nanoparticles was done in an USP XXIV dissolution apparatus (Lab Electronics, Mumbai, India). Accurate amounts of each SGN sample (equivalent to 100 mg of SGN) was placed in 900 mL of dissolution medium with pH 1.2 (simulated gastric fluid) for 2 h then the pH of the medium was converted to 6.8 (simulated intestinal fluid) for 10 h. Throughout the experiment the dissolution medium was kept at 37 ± 0.5 °C with stirring at 50 rpm. At different time intervals, samples of 5 mL were withdrawn and substituted with equivalent volumes of fresh medium to maintain the sink condition [29,30]. The collected samples were subjected to spectrophotometric analysis after appropriate dilution at 267 nm. The experiment was done for each formulation in triplicate and mean ± SD was calculated. 

#### 2.2.6. Optimization of Formulation Variables

Box-Behnken design, using Design-Expert 10 software, was employed to determine the optimum level of three formulation factors which develop SGN–TC nanoparticles with the desired response. The target of the optimization process was to minimize the particle size (nm), maximize the entrapment efficiency (%), and sustained drug release Q_12h_ (%). The Box-Behnken model was constructed using the following Equation (2):

(2) $$Y_{0}=b_{0}+b_{1}A+b_{2}B+b_{3}C+b_{12}AB+b_{13}AC+b_{23}BC+b_{23}BC+b_{11}A^{2}+b_{22}B^{2}+b_{33}C^{2}$$

where Y is the response; b_0_ is the intercept; b_1_ to b_23_ are the regression coefficients; and X_1_, X_2_, and X_3_ are the formulation factors [31]. 

#### 2.2.7. Study of the Effect of TC Concentration on the Properties of SGN–TC Nanoparticles

Three formulations of SGN-TC nanoparticles were prepared using the previously mentioned method with different drug polymer ratios, (1:1), (1:2), and (1:3). The prepared formulations were evaluated for in vitro mucoadhesive study and in vitro drug release. The best formulation was subjected for infra-red spectroscopy (IR), particle size analysis, zeta potential measurement, and transmission electron microscopy (TEM).

##### Mucoadhesion Study of SGN–TC Nanoparticles 

The mucoadhesive properties of the SGN-TC nanoparticles were evaluated using an in vitro wash-off test [32]. A piece of rat intestinal mucosa (2 cm wide and 2 cm long) was placed in a glass slide (3 in. long and 1 in. wide) using thread. One hundred grams of each SGN–TC nanoparticle formulation were placed individually on the wetted intestinal mucosa and allowed to hydrate for 30 s. The intestinal mucosa for each sample was transferred to USP 24 disintegration tester where the vessel of the disintegration tester was filled with 1000 mL of disintegration medium at 37 ± 0.5 °C. The disintegration medium was adjusted to pH 1.2 (simulated gastric fluid) for 2 h then the pH of the medium increased to pH 6.8 (simulated intestinal fluid) up to 10 h. At the end of each hour up to 10 h, the disintegration apparatus was stopped and the percentage of remaining particles adhered to the intestinal mucosa was calculated.

##### In Vitro Release Study

The release study for F1, F2, F3, and free SGN was done as described previously in this manuscript. The release data obtained for the three formulations were exposed to the Ritger and Peppas model to determine the mechanism of drug release [33]. The initial 60% cumulative release data were used to determine the diffusion exponent(‘n’) by using Equation (3):

(3) $$M_{t}/M_{\infty }={K\;t}^{n}$$

where M_t_ is the amount of released drug at time t, M_∞_ is the nominal total amount of drug released, K is the kinetic constant, and ‘n’ the diffusion exponent that is used to characterize the release mechanism. An ‘n’ value less than 0.43 indicates Fickian release, while an ‘n’ value ranging from 0.43 to 0.85 indicates non-Fickian release, and an ‘n’ value higher than 0.85 indicates case-II transport that includes polymer dissolution and expansion of polymeric chains.

##### Infrared (IR) Spectroscopy

The possibility of drug interaction with other ingredients in the prepared nanoparticles was determined by IR spectroscopy. Chitosan, TC, SGN, and the SGN–TC nanoparticles (F3) were characterized by infrared spectroscopy. Each sample was compressed into a disc with potassium bromide (KBr) then subjected to IR spectroscopy using a Thermo Scientific Nicolet IR 200 spectrometer (Thermo Nicolet, Madison, WI, USA) [34,35]. Each compressed sample was scanned from 4000 cm^−1^ to 400 cm^−1^ [36].

##### Particle Size and Zeta Potential Analysis 

The particle size and zeta potential determination of SGN–TC nanoparticles (F3) was carried out using Malvern zeta sizer (Malvern Master Sizer 2000, SM, Malvern, United Kingdom). Liquid samples of F3 were diluted with double distilled water (1:10) and shaken for 30 min to reduce nanoparticle aggregation [37,38]. The particle size and zeta potential of the samples were then measured. 

##### Determination of the Surface Morphology of SGN–TC Nanoparticles 

The surface morphology of SGN–TC nanoparticles (F3) was examined using a transmission electron microscope (JTEM model 1010, JEOL^®^, Tokyo, Japan). One drop of F3 was placed on a collodion-coated copper grid after suitable dilution with double distilled water [39,40]. The sample was allowed to dry and then stained with a uranyl acetate solution. After complete drying of the stain, the sample was examined by TEM (acceleration voltage, 100 kV) [31,41].

#### 2.2.8. In-Vivo Study 

Male Wistar rats (230–250 g) were obtained from the Central Animal House of C.L. Baid Metha College of Pharmacy, Chennai, India. The animals were kept under standard laboratory conditions, with the temperature at 25 ± 1 °C and relative humidity (55% ± 5%). The animals were accommodated in polypropylene cages, four per cage, with free access to standard laboratory diet (Lipton feed, Mumbai, India) and water ad libitum. The protocol of study was approved by the Institutional Animal Ethics Committee, College of Pharmacy, Chennai, India (approval number 02/321/PO/Re/S/01/CPCSEA).

##### Induction of Diabetes and Experimental Groups 

Diabetes was induced in rats by intraperitoneal injection of 50 mg⁄kg streptozotocin (STZ; Sigma, St Louis, MO, USA) dissolved in 0.1 mol ⁄L sodium citrate buffer, pH 4.5 [42]. After 72 h of induction of diabetes, blood glucose level was evaluated. Rats with blood glucose levels >250 mg/dL were used to complete the in vivo study. Animals were divided into five groups (6 rats per group) and treated with the following treatments. Group I: diabetic control group and treated with the vehicle, Group II: diabetic group treated with free SGN (10 mg/kg; po), Group III: diabetic group treated with SGN–TC nanoparticles (F1) (10 mg/kg; po), Group IV: diabetic group treated with SGN–TC nanoparticles (F2) (10 mg/kg; po), and Group V: diabetic group treated with SGN–TC nanoparticles (F3) (10 mg/kg; po). Blood samples were collected from a retro orbital puncture at predetermined time intervals (at the end of each hour for 48 h), and were analyzed for blood glucose by glucose oxidase and peroxidase (GOD/POD) methods using a commercial glucose kit. The following Equation (4) was used to calculate the relative pharmacological availability (PA %).

(4) $$\mathrm{PA}\;\%=\left[\frac{{\mathrm{AAC}}_{\mathrm{NPs}}}{{\mathrm{AAC}}_{\mathrm{SGN}}}\right]\times \left[\frac{{\mathrm{Dose}}_{\mathrm{SGN}}}{{\mathrm{Dose}}_{\mathrm{NPs}}}\right]\times 100$$

where, PA is the pharmacological availability of the SGN, and AAC is the area above the curve. The average blood glucose levels were measured for each group and plotted versus time and the AACs were calculated using the trapezoid rule. 

#### 2.2.9. Statistical Analysis 

The results of the in vivo study were subjected to statistical analysis using the GraphPad Prism statistical package. Student’s *t*-test was used and the difference was considered significant at *p* values < 0.05. 

## 3. Results and Discussion 

### 3.1. Optimization of Formulation Factors of SGN Loaded TC Nanoparticles

In this work, a Box-Behnken design using Stat-Ease design expert software version no.10 was used for preparation of SGN loaded TC nanoparticles for targeting diabetes mellitus. As represented in Table 1, the study was designed to determine the effect of three factors, TC concentration (X_1_), TPP concentration (X_2_), and SGN concentration (X_3_) on the results of particle size (Y_1_), entrapment efficiency (Y_2_), and drug release (Y_3_) of the SGN–TC nanoparticles. The preparation of SGN-TC nanoparticles was performed using the ionic gelation method. Mahdizadeh Barzoki et al. used a Box-Behnken statistical design for optimization of insulin loaded thiolated chitosan nanoparticles [31]. 

According to the previous design, 17 formulations were prepared and evaluated for the results of particle size (Y_1_), entrapment efficiency (Y_2_), and drug release (Y_3_). The compositions of the 17 formulations of SGN–TC are presented in Table 2.

### 3.2. Study of the Effect of Formulation Factors (X_1_, X_2_, and X_3_) on the Responses (Y_1_, Y_2_, and Y_3_)

The results of particle size (Y_1_), entrapment efficiency (Y_2_), and drug release Q_12hr_ (Y_3_) for all formulations are presented in Table 3. It was found that there were wide variations in the results of three responses for the 17 formulations which indicate that the three factors strongly affect the responses. The effect of the three factors in each response was mathematically calculated through the polynomial equation. 

#### 3.2.1. Effect of the Formulation Factors on the Particle Size (Y_1_)

A goal of this study was to minimize the particle size of the prepared SGN–TC nanoparticles as the particle size affects the rate of drug release. To study the effect of the formulation factors (X_1_, X_2_, and X_3_) on the particle size of prepared SGN–TC nanoparticles, multiple linear regression analysis was done using the Y_1_ Equation (5):

(5) $$\begin{array}{cc} \mathrm{particle}\;\mathrm{size}\;\left(Y_{1}\right) & \\ & =+167.60-16.25\;A-10.62\;B-8.38\;C+7.75\;\mathrm{AB}-12.25\mathrm{AC}\\ & +12.00\;\mathrm{BC}+29.70A^{2}+10.45\;B^{2}+25.45\;C^{2} \end{array}$$

**Figure 1:**
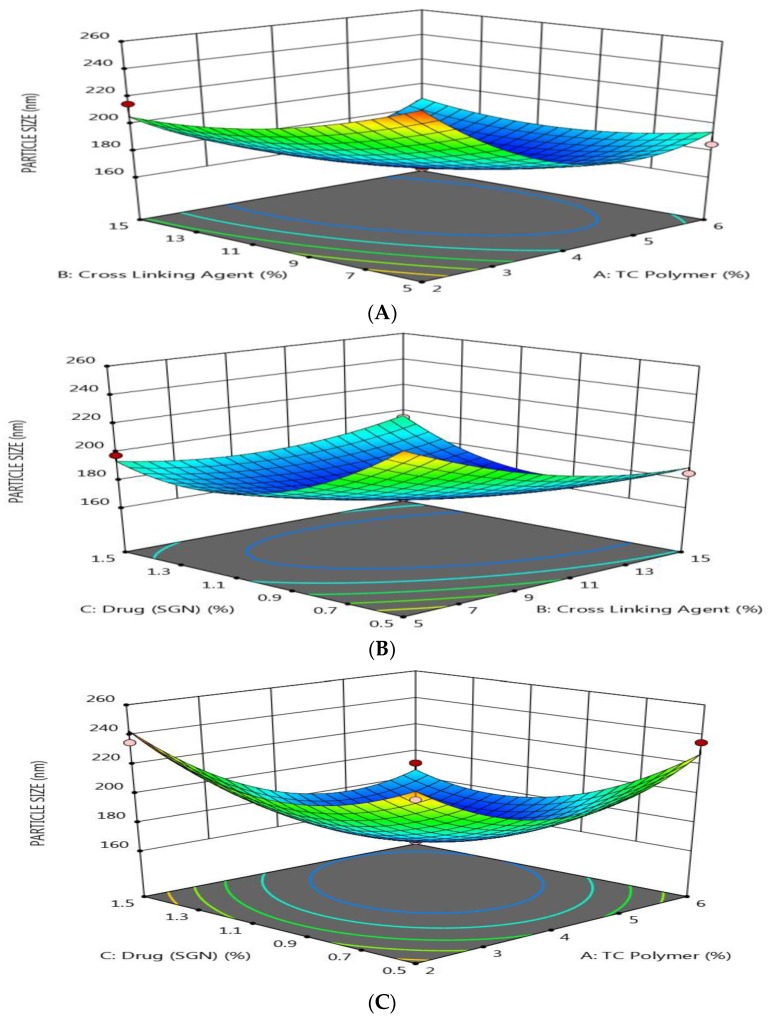
Response surface methodology for the effect of the formulation factors (X_1_, X_2_, and X_3_) on the particles size of SGN–TC nanoparticles. (**A**) Effect of polymer and crosslinking agent concentration on particle size, (**B**) Effect of crosslinking agent and drug concentration on particle size, (**C**) Effect of polymer and drug concentration on particle size.

The response surface plots as shown in Figure 1 show that the particle size of prepared SGN–TC nanoparticles was affected by the formulation factors. 

It was found that the particle size was decreased by increasing the polymer (TC) concentration, the crosslinking agent (TPP) concentration, and drug (SGN) concentration. These results may be attributed to the shrinking of the polymeric layer at high concentrations of TPP as a result of a high degree of crosslinking between the positive charge of amino groups in TC and the negative charge due to PO_4_^−^ ions in TPP [29]. As presented in Table 3, the polydispersity index (PDI) value for all prepared SGN–TC nanoparticles was found to be less than 0.5, which indicates a narrow size distribution [31]. 

#### 3.2.2. Effect of the formulation factors on the Entrapment Efficiency (Y_2_)

A goal of this study was to maximize the entrapment efficiency (EE %) of prepared SGN-TC nanoparticles. To study the effect of the formulation factors (X_1_, X_2_, and X_3_) on the entrapment efficiency of prepared SGN–TC nanoparticles, multiple linear regression analysis was done using polynomial Equation (6):

(6) $$\begin{array}{c} \mathrm{EE}\;\%\;\left(Y_{2}\right)=+47.08+2.89\;A+5.78\;B+2.04C-1.42\mathrm{AB}+2.35\mathrm{AC}-5.98\mathrm{BC}\\ +21.63\;A^{2}-7.14\;B^{2}+9.97\;C^{2} \end{array}$$

**Figure 2:**
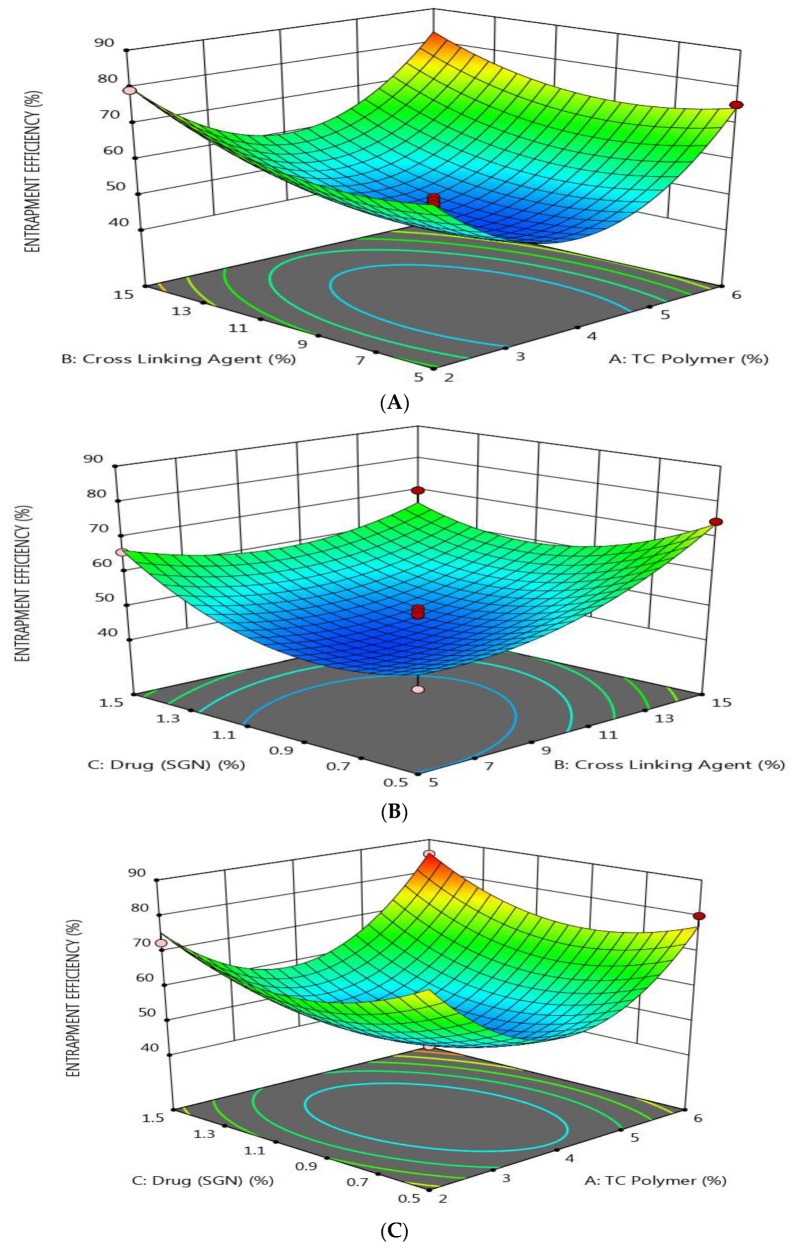
Response surface methodology for the effect of the formulation factors (X_1_, X_2_, and X_3_) on the entrapment efficiency of SGN–TC nanoparticles. (**A**) Effect of polymer and crosslinking agent concentration on entrapment efficiency %, (**B**) Effect of crosslinking agent and drug concentration on entrapment efficiency %, (**C**) Effect of polymer and drug concentration on entrapment efficiency %.

The effect of the three formulation factors on the EE % was explained using response surface plots (Figure 2). It was found that the EE % was increased as the concentration of TC, TPP, and SGN increased. This may be due to the rigid structure of prepared nanoparticles, due to the crosslinking between the TC polymer and TPP, which decrease the leakage of the drug [43]. This result was in full agreement with an earlier study which revealed that the formation of stronger disulfide bonds between TC and PO_4_^−^ ion crosslinking indicated high drug entrapment efficiency [44].

#### 3.2.3. Effect of the Formulation Factors on Drug Release (Y_3_)

The drug release study was conducted in simulated gastric fluid for 2 h and then in simulated intestinal fluid for 10 h as the SGN has an absorption window in the stomach. 

A goal of this study was to decrease and prolong the drug release of SGN-TC nanoparticles. The effect of the formulation factors on drug release was more complex. To study the effect of the formulation factors (X_1_, X_2_, and X_3_) on the drug release of prepared SGN–TC nanoparticles, multiple linear regression analysis was done using polynomial Equation (7):

(7) $$\begin{array}{l} {\mathrm{Drug}\;\mathrm{Release}\;Q}_{12h}\;\left(Y_{3}\right)\\ \;=+93.1-1.75A+2.13B-3.12C-5.75\mathrm{AB}+6.25\mathrm{AC}-0.5\mathrm{BC}\\ \;-20.55A^{2}-15.3B^{2}-9.8C^{2} \end{array}$$

**Figure 3:**
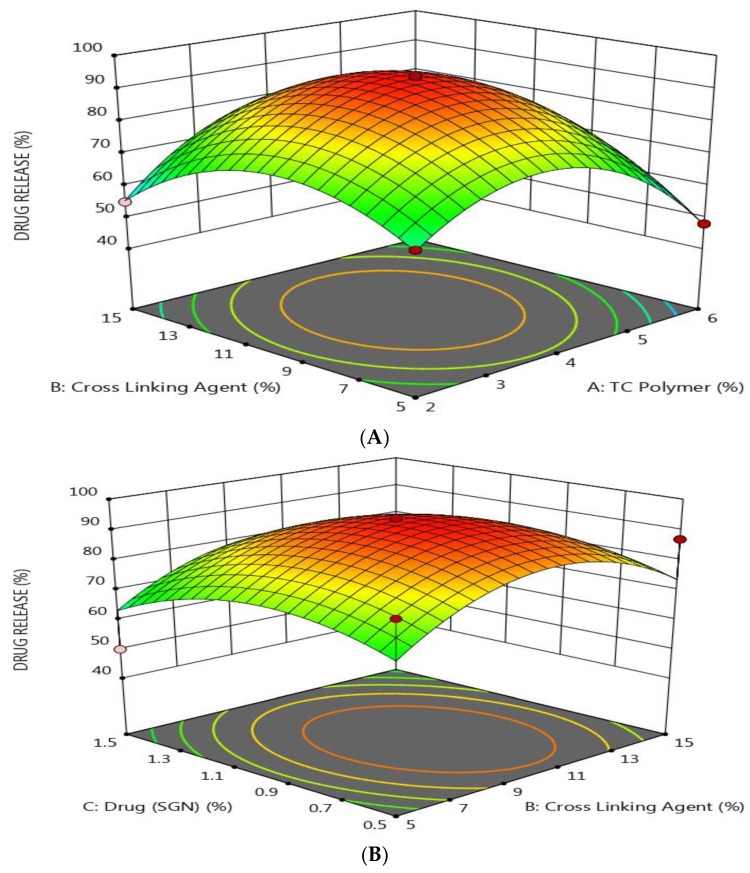

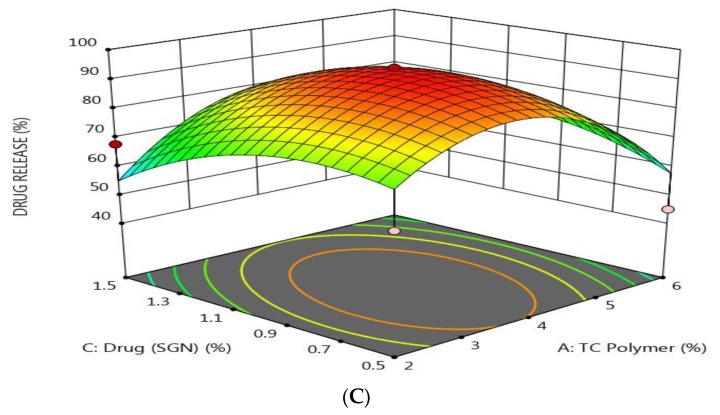
Response surface methodology for the effect of the formulation factors (X_1_, X_2_, and X_3_) on the drug release of SGN–TC nanoparticles. (**A**) Effect of polymer and crosslinking agent concentration on drug release %, (**B**) Effect of crosslinking agent and drug concentration on drug release %, (**C**) Effect of polymer and drug concentration on drug release %.

As shown by response surface methodology in Figure 3, it was found that the drug release was decreased by increasing the concentration of both TC and SGN and by decreasing the level of TPP. These results indicated that there was a direct relationship between the drug release and TPP concentration and an inverse relationship with both TC and SGN concentration. The results may be attributed to the fact that as the TC polymer increased, the diffusion path length increased and drug release was retarded [45]. 

### 3.3. Optimization of Formulation Factors

Statistical analysis based on ANOVA for the response surface quadratic model is presented in Table 4. The P value for the model is less than 0.001, which indicates that it is a significant and desirable model. The large “model F-value” could occur due to noise in the experiments. The “lack of fit F-value” of 3.62 implies that lack of fit is not significant relative to pure error. Thus, it is possible to quantitatively judge if the model represents the observations satisfactorily.

The numerical optimization was done using the Box-Behnken design to determine the optimum level of formulation factors for developing SGN–TC nanoparticles with the desired value of responses.

The goal of the optimization process was to minimize the particle size of prepared SGN–TC nanoparticles, maximize the EE%, and minimize (prolong) the drug release. So, a constraint for each response was selected to obtain the optimum level of each formulation factor which gives an optimized formulation using Design-Expert 10 software. The optimum levels of formulation factors for an optimized formulation based on the Box-Behnken design were 2% *w*/*v* of TC polymer, 12.21% *w*/*v* of TPP, 1% *w*/*v* of SGN with predicted values of 181.02 nm for particle size, 76.68% for EE%, and 69.49% for drug release (Q12 h). The optimized formulation was prepared using the ionic gelation method and the actual values of the responses were 179.64 ± 8.22 nm for particle size, 78.23 ± 3.46% for EE%, and 71.96 ± 3.14% for drug release (Q12 h). The actual values of responses were found to be close to the predicted values which indicated the validity of the Box-Behnken design. 

### 3.4. The Effect of TC Concentration on Mucoadhesive Properties and in Vivo Efficacy of SGN–TC Nanoparticles

Based on the results of the Box-Behnken design it was found that the concentration of TC polymers had the highest effect on the results of drug release. So, three new formulations were prepared using the same procedure mentioned before with different concentrations of TC to study the effect on mucoadhesive properties and the in vivo efficacy of SGN–TC and the compositions of the formulations are presented in Table 5.

### 3.5. In Vitro Mucoadhesive Study

The mucoadhesion study for SGN–TC nanoparticles was done at pH 1.2 for 2 h and at pH 6.8 up to 10 h. The percentage of mucoadhesivity of the nanoparticles is shown in Table 6. The percentage of SGN–TC nanoparticles (F1) adhering to tissue ranged from 74 ± 5.3 after 1 h to 6 ± 1.4% after 10 h. The percentage of adhering nanoparticles ranged from 83 ± 4.7 after 1 h to 14 ± 2.0 after 10 h in formulation (F2). For (F3) the percentage of nanoparticles adhered to the tissue ranged from 91 ± 3.0 after 1 h to 21 ± 1.2 after 10 h. From the results of mucoadhesive study, it was found that the percentage of nanoparticles adhered to intestinal tissue increased as the polymer concentration (TC) increased. These results may be due to the increase in the concentration of thiolate anions caused by increasing the concentration of polymer (TC) which resulted in improvement the mucoadhesion of SGN–TC nanoparticles to the mucus gel layer based on covalent attachment [5]. These results were in good agreement with Zhou et al. who found that thiolated nanoparticles increased the adhesion of insulin to the mucous membrane when taken by the oral route [46].

### 3.6. In Vitro Release of SGN from Prepared SGN–TC Nanoparticles

**Figure 4:**
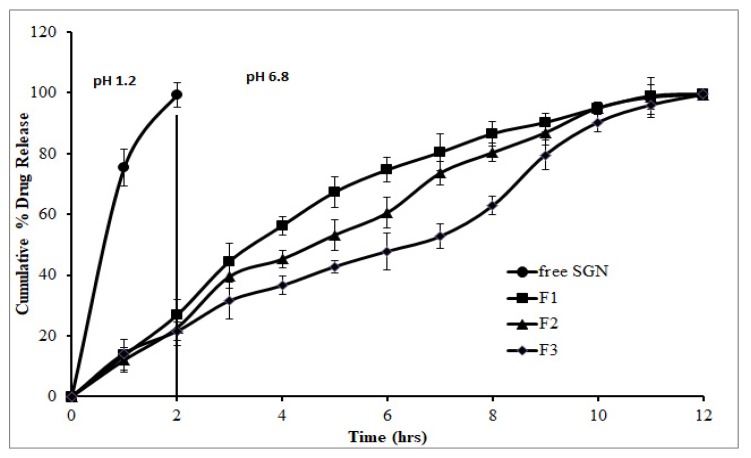
In vitro release of SGN from prepared SGN–TC nanoparticles.

The in vitro drug release profiles of SGN–TC nanoparticles and free SGN are shown in Figure 4. The release of SGN from nanoparticles showed prolonged drug release in comparison to the free SGN which showed fast release. It was found that there was an inverse relationship between the polymer concentration and the drug release. All the formulations were found to retard the drug release up to 12 h whereas free SGN was observed releasing quickly within 1 h. This might be due to the increased swelling ability of the TC polymer which resulted in increasing the diffusion path length for the drug, hence retarding the drug release from the nanoparticles [47]. 

### 3.7. In Vitro Release Kinetics

**Figure 5:**
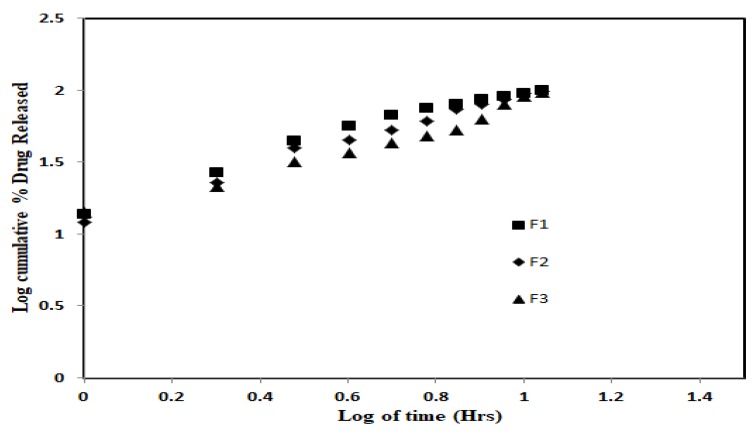
In vitro release kinetics of SGN from SGN-TC nanoparticles (F1–F3).

The in vitro release kinetics for the prepared SGN-TC nanoparticle were evaluated to obtain the suitable behavior for the release of SGN from nanoparticles. The in vitro release data of F1–F3 were subjected to zero-order kinetics, first-order kinetics, and the Higuchi diffusion model. It was found that the release of SGN from TC nanoparticles followed the Higuchi model which revealed the highest correlation coefficient value (*r*^2^ = 0.99). The results indicated that the release of SGN from mucoadhesive TC nanoparticles was diffusion controlled which represents one tool of achieving sustained drug release. The mechanism of drug release from polymeric drug delivery systems usually follows the Higuchi diffusion model which was described by Fickian diffusion. In the case of the preparations containing swelling polymer, there is another mechanism for drug release besides diffusion. This process includes relaxation of polymer chains and imbibition and retention of water leading to swelling of the polymers and changing them from their initial glassy to rubbery states [44]. As a result of the swelling process, the release data were further treated with the Ritger and Peppas equation (Power law). The release data of SGN–TC nanoparticles (F1–F3) were treated with the Power law to determine the release mechanism. The initial 60% cumulative drug release data were used to determine the diffusion exponent ‘n’:

$$M_{t}/M_{\infty }=K\;t^{n}$$

where M_t_ is the amount of drug released at time t, M_∞_ the nominal total amount of drug released, K the kinetic constant, and *‘n*’ the diffusion exponent which is used to characterize the release mechanism. According to Ritger and Peppas equation, ‘n’ takes values in the range of 0.45–0.89 for the anomalous release mechanism. The value of ‘n’ with a regression coefficient for the prepared SGN–TC nanoparticles was found to be 0.874 for F1, 0.818 for F2, and 0.791 for F3, which gave an indication of the anomalous release mechanism (Figure 5). The anomalous diffusion mechanism of drug release revealed both diffusion and swelling controlled drug release from SGN–TC nanoparticles.

### 3.8. Fourier Transform Infrared Spectroscopy (FTIR) 

**Figure 6:**
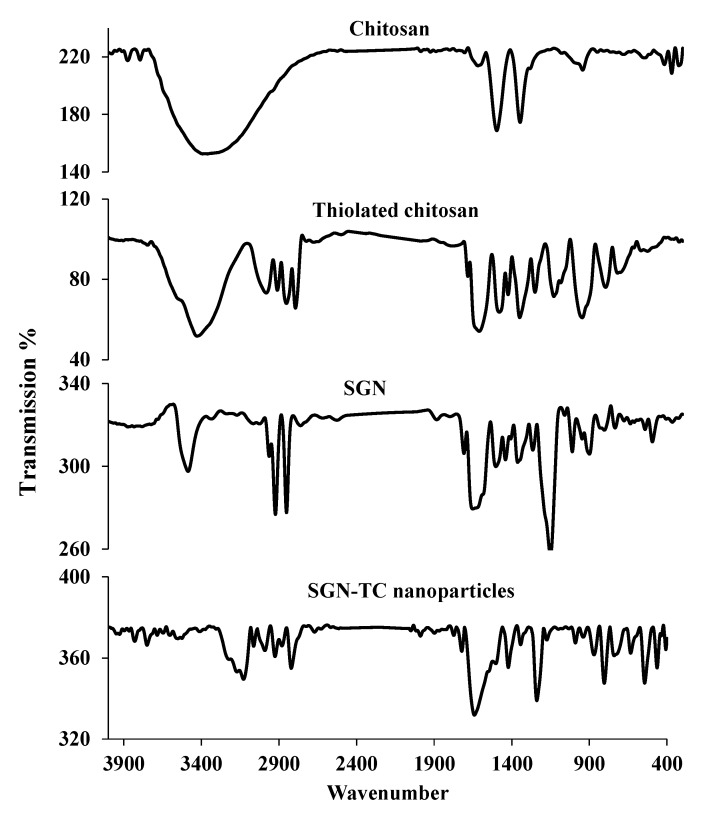
Fourier transform infrared spectroscopy (FTIR) of chitosan, thiolated chitosan, SGN, and SGN–TC nanoparticles.

The FTIR spectrums of chitosan, TC, SGN, and SGN–TC nanoparticles are shown in Figure 6. The IR spectrum of chitosan showed characteristic peaks at 3365 cm^−1^ (N–H stretching and –H), 1665 cm^−1^ (C=O), 1549 cm^−1^ (N–H), and 1404 cm^−1^ (CH–OH). The IR spectrum of TC showed characteristic peaks at 2496 cm^−1^ (–SH stretching), 808 cm^−1^ (S–S bisulfide bond), and 1245 cm^−1^ (CSH stretching) which did not appear in the IR spectrum of chitosan. These results gave a good indication about presence of thiol groups of thioglycolic acid in TC. The same results were obtained by Zhang et al. who prepared thiolated chitosan nanoparticles [48]. The IR spectrum of SGN showed characteristic peaks at 3401.11 cm^−1^ (amine functional group), 3210.11 cm^−1^ (C–H stretching), 1799.89 cm^−1^ (C=O group), 1514.24 cm^−1^ (N–H bending) of primary amides, and 1489.36 cm^−1^ (N–H bending) of secondary amides. The IR spectrum of SGN–TC nanoparticles (F3) showing all characteristic peaks of SGN indicated that there was no interaction between SGN and TC polymers. These results were in full agreement with SreeHarsha et al. who prepared mucoadhesive nanoparticles of sitagliptin using thiolated chitosan [3]. 

### 3.9. Particle Size and Zeta Potential of SGN–TC Nanoparticles

**Figure 7:**
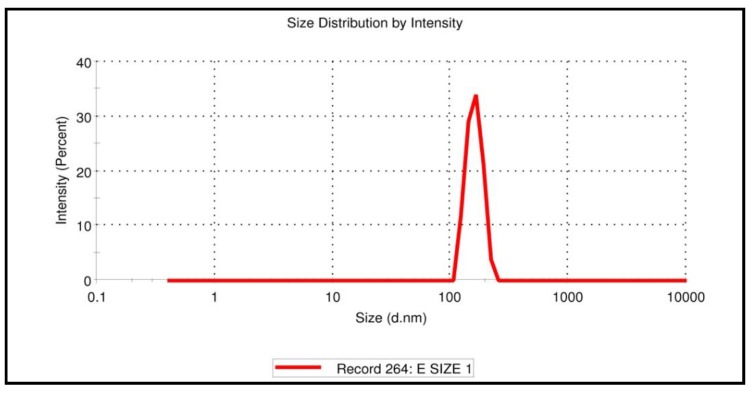
Size and size distribution curve of SGN–TC nanoparticles (F3).

The particle size of SGN–TC nanoparticles (F3) was measured using laser light diffraction techniques. It was found that negatively charged phosphate ion of the TPP had firmly integrated on the amino group (cationic charge) of TC and the SGN–TC nanoparticles were almost spherical in shape with an average diameter of less than 160.3 ± 10.9 nm with high reproducibility. As shown in Figure 7, the particle size distribution curve was unimodal with a narrow range.

**Figure 8:**
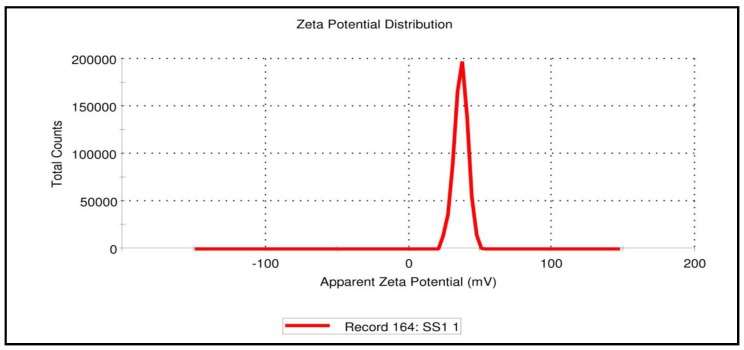
Zeta potential of SGN–TC nanoparticles (F3).

As shown in Figure 8, zeta potential measurement of SGN–TC nanoparticles (F3) was found to be 36 mV with positive charge. The positive charge of the prepared nanoparticles may be due to the unreacted amino group in the chitosan polymer [49]. An earlier study revealed that positively charged particles could increase electrostatic interactions with the negatively charged mucin present in the mucosal surface, thus leading to improved bioavailability and reduced side-effects [50]. The result was in full accordance with Mahdizaden et al. who found that the values of zeta potential of the prepared insulin thiolated chitosan nanoparticles were positive [51].

### 3.10. The Surface Morphology of SGN–TC Nanoparticles 

**Figure 9:**
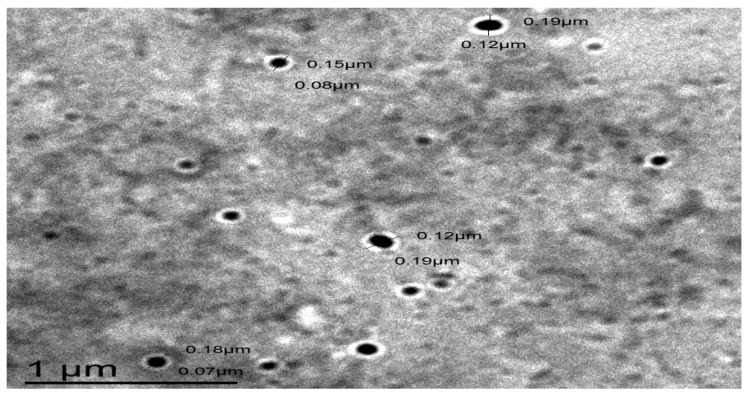
A transmission electron microscope (TEM) image of SGN–TC nanoparticles (F3).

The TEM image of SGN–TC nanoparticles (F3), as shown by Figure 9, proved that the prepared SGN–TC nanoparticles possessed a spherical shape and nano size. The spherical shape of SGN–TC nanoparticles revealed the successful crosslinking between TC polymers and TPP. The crosslinking occurred via intra and intermolecular interactions between the amine groups of TC (positively charged) and phosphate ions of TPP (negatively charged) were responsible for the ionic gelation which could provide structural integrity of the nanoparticles [52]. Anitha et al. found that the prepared thiolated chitosan nanoparticles of 5-flurouracile were spherical in shape [53].

### 3.11. Oral Efficacy of SGN–TC Nanoparticles 

**Figure 10:**
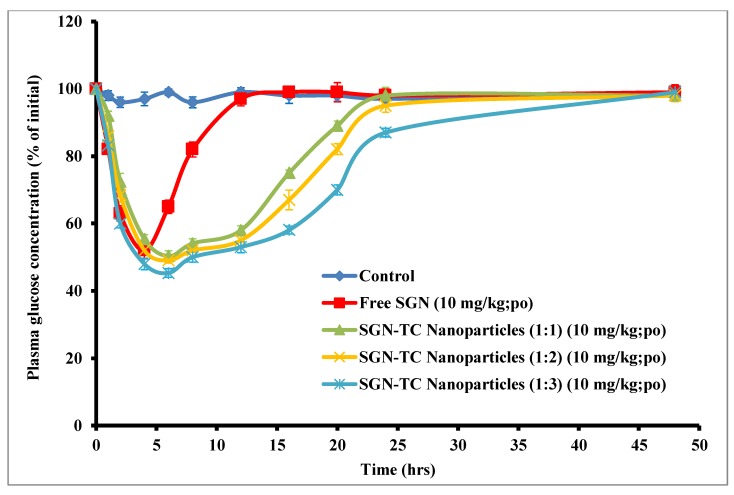
In vivo hypoglycemic efficacy of SGN–TC nanoparticles in comparison with free SGN.

The in vivo study of prepared SGN–TC nanoparticles (F1–F3) was done in STZ-induced diabetic rats in comparison to free SGN. As shown by Figure 10, SGN–TC nanoparticles gave a prolonged antidiabetic effect compared to free SGN. The free SGN exhibited a reduction in blood glucose level and the antidiabetic effect was maintained for only 6 h, while the hypoglycemic effect of prepared SGN–TC nanoparticles was prolonged for more than 24 h. The prolonged effect of SGN–TC nanoparticles may be attributed to the mucoadhesive properties of TC nanoparticles which resulted in adhesion of nanoparticles to the mucus gel layer [54]. The prolonged release of SGN–TC nanoparticles is significantly more effective than the immediate release of free SGN in reduction of blood glucose levels. 

The pharmacokinetic parameters of glucose levels after administration of all treatments are shown in Table 7. The mucoadhesive SGN–TC nanoparticles F1, F2, and F3 produced minimum glucose levels of 50.32% ± 3.01%, 49.05% ± 2.55%, and 45.31% ± 1.91% at 6 h respectively. These results could be attributed to the longer residence time of the SGN in the gastrointestinal tract using the TC nanoparticle carrier system, which keeps the drug in the absorption site for a longer time. Orally administered free SGN with the same dose showed a slight reduction in blood glucose level due to poor permeability and residence time in gastrointestinal tract (GIT). The relative pharmacological efficacy of mucoadhesive SGN–TC nanoparticles was 349.9% ± 6.0%, 359.3% ± 3.8%, and 474.05% ± 3.2% for F1, F2, and F3 respectively which was significantly higher than the efficacy of free SGN. This significant increase in the pharmacological efficacy of SGN in the TC nanoparticle formulations (*p* < 0.05) could be related to the mucoadhesion properties and permeability enhancing effects of TC polymers [55]. These results were in good agreement with Sudhakar et al. who prepared insulin loaded thiolated chitosan nanoparticles and found that the efficacy of insulin against diabetes induced rats was higher than the free insulin [42].

## 4. Conclusions

The authors concluded that SGN was successfully prepared as SGN–TC nanoparticles using the ionic gelation method for treatment of type II diabetes mellitus. The prepared SGN–TC nanoparticles showed high entrapment efficiency, uniform particle size, and prolonged drug release. Thiolated chitosan concentration had a great effect on the rate of SGN release and the mucoadhesion properties of nanoparticles. The mucoadhesion rate increased when concentration of TC polymers was increased. TC nanoparticles had the ability to control and prolong the systemic absorption of SGN. SGN–TC nanoparticles significantly reduced plasma glucose level compared to free SGN in STZ-induced diabetic rats. The TC nanoparticles are useful carriers for oral controlled delivery of drugs with various therapeutic uses.

## Figures and Tables

**Table 1:** The formulation factors and responses of Box-Behnken design for sitagliptin thiolated chitosan (SGN–TC) nanoparticles.

			Level Used	
Factor	Name	Units	Low (−1)	High (+1)
A: X_1_	Thiolated Chitosan (TC)	(% *w*/*v*)	2	6
B: X_2_	Tripolyphosphate (TPP)	(% *w*/*v*)	5	15
C: X_3_	Sitagliptin (SGN)	(% *w*/*v*)	0.5	1.5
**Response**	**Name**	**Units**	**Goal**	
Y_1_	Particle Size	Nm	Minimize	
Y_2_	Entrapment Efficiency (EE)	%	Maximize	
Y_3_	Drug Release (Q_12h_)	%	Minimize	

**Table 2:** The designed SNG–TC nanoparticles according to the Box-Behnken experimental design.

Run	X_1_	X_2_	X_3_
	A: Polymer (TC)	B:Cross Linking Agent (TPP)	C:Drug (SGN)
	% (*W*/*V*)	% (*W*/*V*)	% (*W*/*V*)
1	6	10	0.5
2	4	10	1
3	4	10	1
4	2	10	0.5
5	4	15	1.5
6	4	10	1
7	4	15	0.5
8	2	10	1.5
9	6	10	1.5
10	4	5	1.5
11	4	10	1
12	2	15	1
13	6	15	1
14	6	5	1
15	4	10	1
16	2	5	1
17	4	5	0.5

**Table 3:** The measured responses of prepared SGN–TC nanoparticles according to the Box-Behnken experimental design.

Run	Y_1_	Y_2_	Y_3_	
	Particle Size nm	Entrapment Efficiency %	Drug Release (Q_12h_) %	Polydispersity Index (PDI)
1	235 ± 5.26	80.11 ± 2.14	45.25 ± 1.25	0.465 ± 0.07
2	167 ± 3.54	48.5 ± 0.95	93.13 ± 1.83	0.258 ± 0.02
3	166 ± 6.58	49.51 ± 2.85	92.32 ± 0.85	0.321 ± 0.06
4	230 ± 8.47	76.44 ± 1.29	61.61 ± 1.27	0.128 ± 0.03
5	195 ± 7.15	69.9 ± 1.64	54.11 ± 2.85	0.267 ± 0.09
6	168 ± 3.82	42.66 ± 0.86	94.21 ± 0.97	0.436 ± 0.05
7	185 ± 6.24	74.53 ± 1.23	87.37 ± 1.28	0.239 ± 0.06
8	235 ± 7.36	72.56 ± 2.46	68.39 ± 2.39	0.365 ± 0.02
9	191 ± 3.24	85.63 ± 3.12	77.47 ± 2.44	0.487 ± 0.01
10	198 ± 5.34	65.82 ± 1.98	50.02 ± 1.29	0.195 ± 0.04
11	169 ± 6.17	47.21 ± 1.56	93.45 ± 2.36	0.466 ± 0.07
12	215 ± 3.78	79.21 ± 2.94	55.12 ± 0.86	0.369 ± 0.06
13	185 ± 6.82	79.55 ± 3.88	63.67 ± 1.27	0.241 ± 0.03
14	185 ± 2.67	75.32 ± 0.97	48.20 ± 0.83	0.456 ± 0.08
15	167 ± 8.75	47.52 ± 1.25	93.81 ± 2.63	0.366 ± 0.07
16	246 ± 5.22	69.32 ± 2.94	63.31 ± 1.14	0.211 ± 0.09
17	236 ± 4.95	46.52 ± 1.82	81.89 ± 1.37	0.299 ± 0.01

**Table 4:** ANOVA for the quadratic model developed for the optimization of SGN–TC nanoparticles.

Source	Sum of Squares	DF	Mean Square	F Value	*p* Value
Model	3366.61	9	374.07	25.48	<0.0001
Residual	102.78	7	14.68	—	—
Lack of fit	75.11	3	25.04	3.62	0.1230
Pure error	27.67	4	6.92	—	—
Corr. total	3469.38	16	—	—	—

**Table 5:** The compositions of SGN–TC nanoparticles based on different drug polymer ratios.

Formulation Code	SGN:TC Ratio
F1	1:1
F2	1:2
F3	1:3

**Table 6:** Percentage of nanoparticles adhering to tissue at pH 1.2 and pH 6.8.

Formulation Code	pH 1.2		pH 6.8			
	1 h	2 h	4 h	6 h	8 h	10 h
**F1**	74 ± 5.3	59 ± 3.7	53 ± 4.1	49 ± 3.9	23 ± 2.5	6 ± 1.4
**F2**	83 ± 4.7	72 ± 4.2	64 ± 5	55 ± 3.4	34 ± 2.8	14 ± 2.0
**F3**	91 ± 3.0	83 ± 2.9	70 ± 3.5	62 ± 2.8	42 ± 1.6	21 ± 1.2

**Table 7:** The relative pharmacological efficacy of SGN–TC nanoparticles.

Parameters	Free SGN	SGN–TC Nanoparticles (1:1)	SGN–TC Nanoparticles (1:2)	SGN–TC Nanoparticles (1:3)
SGN dose (mg)	10 mg/kg	10 mg/kg	10 mg/kg	10 mg/kg
Minimum glucose level in % of the initial level	52.06 ± 2.86	50.32 ± 3.01	49.05 ± 2.55	47.09 ± 1.91
Time point of minimum glucose level (h)	4	6	6	6
AAC_0→48_	776.5 ± 8.34	2686 ± 6.2 *	2790 ± 7.5 *	3681 ± 5.7 *
Relative pharmacological efficacy (PA %)	-	349.9 ± 6.0 *	359.3 ± 3.8 *	474.05 ± 3.2 *

Results are expressed as (mean ± SD, *n* = 6). * *p* < 0.05
